# Supplementary material for: Patterns of Intron Gain and Loss in Fungi
Source: PLoS Biol. 2004 Nov 30;2(12):e422. doi: 10.1371/journal.pbio.0020422 (PMC532390; doi:10.1371/journal.pbio.0020422)
Supplement: Table S1 — Also available at http://genes.mit.edu/NielsenEtAl/. (4.3 MB ZIP). [file pbio.0020422.st001.zip › NielsenEtAl/html/331.html]

AN4332.1.NCU02468.1.MG03090.1.FG09423.1


```
 CLUSTAL W (1.82) Multiple Sequence Alignments - Introns Inserted


Sequence 1: NCU02468.1	509 aa
Sequence 2: FG09423.1	521 aa
Sequence 3: MG03090.1	475 aa
Sequence 4: AN4332.1	429 aa
Alignment Length: 555 aa
Number Identitical Residues: 132 aa
Alignment Score (without introns) 8717


MG03090.1 	------------MPPDRK------------------------------------------
NCU02468.1	--------MGDSHTQSSSNGPDS--------RMRRNTATSSVPSSGITS---SSSAALPP
FG09423.1 	MSIDPVMQVEDKIAPGPSANNDKPAVEETIIRQTIITTTEDEAHEHIAASNGSASAATSP
AN4332.1  	------------------------------------------------------------
          	                                                            

MG03090.1 	----------QKRESAPAR--PPYSQ--------LRRTIKKEDVAMSGFTRWCVKHQIG1
NCU02468.1	QRRTVKR---EKSGNPKNMNGPLYMQRNEN-IVLVRRVKRKGEGPLKQLTRWFVENQIG1
FG09423.1 	TTGAASRPRLKKDNSTPNMNGPLYMQTAGNKTVLVRRLKRKEESTWKHLSRWFVENQIG1
AN4332.1  	---------MAKDTTLPAQSVTELNA--------CVTTSSHRPVKDTTFREWVVSNQIG1
          	           *  .    . .                  :     . : .* *.:*** 

MG03090.1 	LSMNLLALLALTHVCFPKSHKYTTKFFSQSYYNPRSGEYGIGKDDYYQIFFYMTIFTALR
NCU02468.1	FAFNLLALLFLAHGCMPRARSHTGKYFTLSYYNPTTGQYGLGKDDGFLIFFCIILFTGLR
FG09423.1 	LSFNLLALIFLAQTFIPKARDYTQKFFHLSYHNSQSGQYRIGYDDAYFITFCIILFTGLR
AN4332.1  	ISLTTLSMLLAVHHLYPSLSPYTTPFFQLSYYQPSQGVYIQGWDDIYFVISSVFAFTAIR
          	:::. *:::  .:   *    :*  :*  **::.  * *  * ** : :   :  **.:*

MG03090.1 	AGTMDYVLAPLARWWGITKRKTIDRFSEQGYLVLYYAIFWPMGM0YIYCNSDYYMNLTNL
NCU02468.1	AATLEYLFAPYAKSKGISKRKDITRFSEQAWLLIYYSVFWTLGL0YIYWQSPHFFNLREL
FG09423.1 	AATMEYVLAPIGRWQGVDNRKNLTRFSEQAWLMVYYTVFWPWGV0YIYCTSPHYMSMENL
AN4332.1  	AIAMEWVFWPLARWSGL-KRKASIRLAEQGWMWLYYAVFWTVGM0YIWSQSDYWMDFKAI
          	* :::::: * .:  *: :**   *::**.:: :**::**. *: **:  * :::.:  :

MG03090.1 	WTNWPNREVSGLMRVYMLAQLAFWFQQILVINIEERRKDHWQMFAHHVVTIVLITTSWRY
NCU02468.1	WTNWPNRELTGIMKGYMLAQLAFWLQQILVINIEERRKDHWQMFTHHIITICLIYASYRY
FG09423.1 	WTDWPNRELDGIMKAYLLCQWAFWLQQMIVINIEERRKDHWQMFTHHIVTTALIFACYAY
AN4332.1  	WAHWPARGVSGLMKWYLLAQLAFWVQQIFVINIEERRKDHYQMLTHHFITSCLLTSAYVY
          	*:.** * : *:*: *:*.* ***.**::***********:**::**.:*  *: :.: *

MG03090.1 	GYTRVGNLILILMDGVDIVFS0AAKLLKYTGFDTACDVFFGLFMLSWVIARHFVYLTVCW
NCU02468.1	GHTRVGNLILVLMDVVDLFLP0LAKCLKYLGHSSACDFMFVVFMVSWFVARHVLYLTVCY
FG09423.1 	HHTRVGNFILVIMDVVDLFLP0LAKCLKYCGYKKICDVMFGLFVVSWFFARHVLYIAVCW
AN4332.1  	GFYNVSNVVLNLMDIVDLLLP0TAKILKYLKFEMSCNIAFGVFMVVWAISRHIMYPLLCW
          	 . .*.*.:* :** **:.:.  ** ***  ..  *:. * :*:: * .:**.:*  :*:

MG03090.1 	SIYKEAPSVMPDGCFRGPADKIEGPFPVPSDRGLWYIVEPLINNNSLVCFDDPVKWSFLL
NCU02468.1	SVYAHTTSIIPYGCFSGTPGNLKGPFQPPADKGALYLLEPLWNPDGLVCYDDTVKWTFLS
FG09423.1 	SIYSDTPRIMPTGCFKGNNENMIGPLNPPAGWG--YLVEPFINPQGLVCYNETVKWGFLG
AN4332.1  	SIFKDVPAVMPWGCYSGATGELISTNGYP--DRVMHLFSPFLNIDGPICMNRTIKWIFLS
          	*:: ... ::* **: *   :: ..   *      ::..*: * :. :* : .:** ** 

MG03090.1 	SLLFLQGILLVWLVAIFRVAIKVLRGTGAEDTRSDDEEDE----EEDIEADGVTYQKTGA
NCU02468.1	MLLFLQALTIMWFSLIIRVALRVLRGEGADDTRSDDEGCEDVDAEDDEDETEFVYEEA--
FG09423.1 	PLLFLQAITIGWFTMIVRVIVKVLKGGDAEDVRSDDEGGE------EEEEEEFVYEEA--
AN4332.1  	SLLALQVLSIIWFSMVIRVAIGVLRTGNAEDTRSDSEEKD------EEEVDPKSAANG--
          	 ** ** : : *:  :.** : **:  .*:*.***.*  :      : :       :   

MG03090.1 	TLKDAVAGRGGKTTLSDDDNDEPIEEEVGVEDIDFKGWG--RRSGVKRQASSATGVSLPG
NCU02468.1	---------------------QALAEEVGVDELDLKNWAERRRAGHGVGGVTATGVSLPG
FG09423.1 	---------------------QPLEEEVGVEALDLRNWE--RRSGVKK-QASSSGVSLPG
AN4332.1  	---------------------NAIAAEVSSAEW--------RRNGPSS--------TRSR
          	                     :.:  **.            ** *           : . 

MG03090.1 	HSDRKELLGRIGCEKQVD
NCU02468.1	HSDRKELLGRIGCEKQVE
FG09423.1 	HSDRKELLGRIGCEKQVD
AN4332.1  	RSDRKALLGRIGCDTRT-
          	:**** *******:.:.
```
